# Lumbar ossification of the ligamentum flavum reflects a strong ossification tendency of the entire spinal ligament

**DOI:** 10.1038/s41598-023-27650-z

**Published:** 2023-01-12

**Authors:** Kazuha Nakabachi, Tsutomu Endo, Masahiko Takahata, Ryo Fujita, Yoshinao Koike, Ryota Suzuki, Yuichi Hasegawa, Toshifumi Murakami, Katsuhisa Yamada, Hideki Sudo, Mohamad Alaa Terkawi, Ken Kadoya, Norimasa Iwasaki

**Affiliations:** 1 Department of Orthopedic Surgery, Hokkaido University Graduate School of Medicine, Kita-15 Nishi-7, Kita-Ku, Sapporo, Hokkaido 060-8638 Japan; 2 Department of Orthopedics, Hakodate Central General Hospital, 33-2 Hon-Cho, Hakodate, Hokkaido 040-8585 Japan

**Keywords:** Physiology, Bone, Orthopaedics

## Abstract

Patients with ossification of the ligamentum flavum (OLF) in the lumbar spine may be at high risk of developing concomitant ossification of the entire spinal ligament, but the etiology remains unclear. We investigated the propensity for spinal ligament ossification in asymptomatic subjects with lumbar OLF using the data of 595 Japanese individuals receiving medical check-ups, including computed tomography (CT) scanning. The severity of OLF (total number of intervertebral segments with OLF) of the entire spine on CT was quantified using an OLF index. Subjects with OLF were grouped according to this index: localized OLF (n = 138), intermediate OLF (n = 70), and extensive OLF (n = 31). The proportion of subjects with lumbar OLF increased with increasing OLF index (localized 13.7%, intermediate 41.4%, and extensive 70.9%). Multiple regression analysis found that lumbar OLF index was associated with thoracic OLF index, and co-existence of ossification of the posterior longitudinal ligament (OPLL) of the thoracic and lumbar spine. This study showed that subjects with more multilevel lumbar OLF were more likely to develop multilevel thoracic OLF and to have coexisting OPLL. Patients with lumbar OLF may be a distinctive subgroup with a strong tendency to ossification of the entire spinal ligament.

## Introduction

Ossification of the ligamentum flavum (OLF) is a relatively common spinal ligament ossification disease in East Asian countries, including Japan^1–6^. OLF mainly affects the thoracic spine; it rarely affects the lumbar spine (0.3%), and very rarely the cervical spine^7^. While degeneration of ligamentous attachments due to mechanical factors or local spinal instability may be involved in the development of OLF^8–10^, the precise etiology and underlying background of patients with the disease remain unclear.

A tendency toward ossification of the entire spinal ligament contributes to the exacerbation of OLF^11^. Previous epidemiological studies have found that OLF is often coexistent with ossification of the posterior longitudinal ligament (OPLL) of the thoracic spine, which is characterized by the presence of diffuse ossified lesions in the spinal ligaments^12,13^. Our recent study indicated that symptomatic patients with coexisting OLF of the lumbar spine were likely to have severe OLF over the entire spine^14^. Patients with OLF in the lumbar spine may be a distinctive subgroup in which OLF is exacerbated by humoral factors affecting systemic bone metabolism. However, our previous study of symptomatic OLF patients had selection bias and limitations in characterizing patients at high risk of developing OLF. In the current study, we attempted to reduce selection bias and ensure a better understanding of the characteristics of subjects with lumbar OLF by using a large dataset of medical records of asymptomatic subjects based on medical check-ups at a single institution.

No therapeutic approach is available to inhibit the ossification of spinal ligaments or slow the progression of ossified lesions. Identifying the background of patients with different developmental patterns of ossification and distinguishing the causes of spinal ligament ossification may lead to the future detection of a group of patients who can be treated by controlling systemic metabolic abnormalities. Our objective was to confirm the ossification tendency of the entire spine in subjects who have lumbar OLF.

## Methods

### Study design

This retrospective cross-sectional study, which was conducted in accordance with the Declaration of Helsinki (1964), included subjects who underwent health screenings from April 2020 to January 2021. The study was approved by the ethical review board of the Hakodate Central General Hospital (Hakodate Central Gereral Hospital Clinical Trial Center, approval number: 2020-14) and Hokkaido University Hospital (Hokkaido University Hospital Clinical Research Administration Center, approval number: 019-0170). The need for informed consent from patients was waived as this study used retrospective, de-identified data.

### Patients

We utilized a database of 12,390 Japanese subjects from a single institution. Regardless of the presence or absence of symptoms, all subjects had undergone annual or multiannual medical check-ups for the early detection of diseases, including cancer. Subjects included community residents and institutional staff, such as doctors, nurses, nursing assistants, therapists, and office staff. Computed tomography (CT) of the body trunk was performed at the subject's discretion, and the scan range (neck to chest, abdomen to pelvis, and neck to pelvis) was also chosen by the subject. Of the 976 subjects who underwent CT scanning, there were 595 for whom CT could be used to evaluate the cervical spine to the pelvis. The final sample included 239 subjects with OLF (162 men and 77 women) ranging in age from 29 to 77 years who reported no numbness or other symptoms in their limbs on a questionnaire.

### Clinical background and distribution of spinal ligament ossification

Clinical background data were obtained from a database that consisted of physical measurements, including height and weight, and a questionnaire completed by all subjects. The questionnaire included items on the presence of comorbidities such as lifestyle-related diseases and the presence of symptoms, including numbness in the upper and lower limbs. The presence and location (i.e., cervical [C], thoracic [T], and lumbar spine [L]) of spinal ligament ossification, including OPLL, OLF, and ossification of the anterior longitudinal ligament (OALL), were assessed using axial and sagittal reconstruction of CT images of the whole spine. CT was performed using an Aquilion ONE™/GENESIS Edition system (Canon Medical Systems Corporation, Tochigi, Japan).

### Evaluation of the presence OPLL, OLF, and OALL

**Figure 1:**
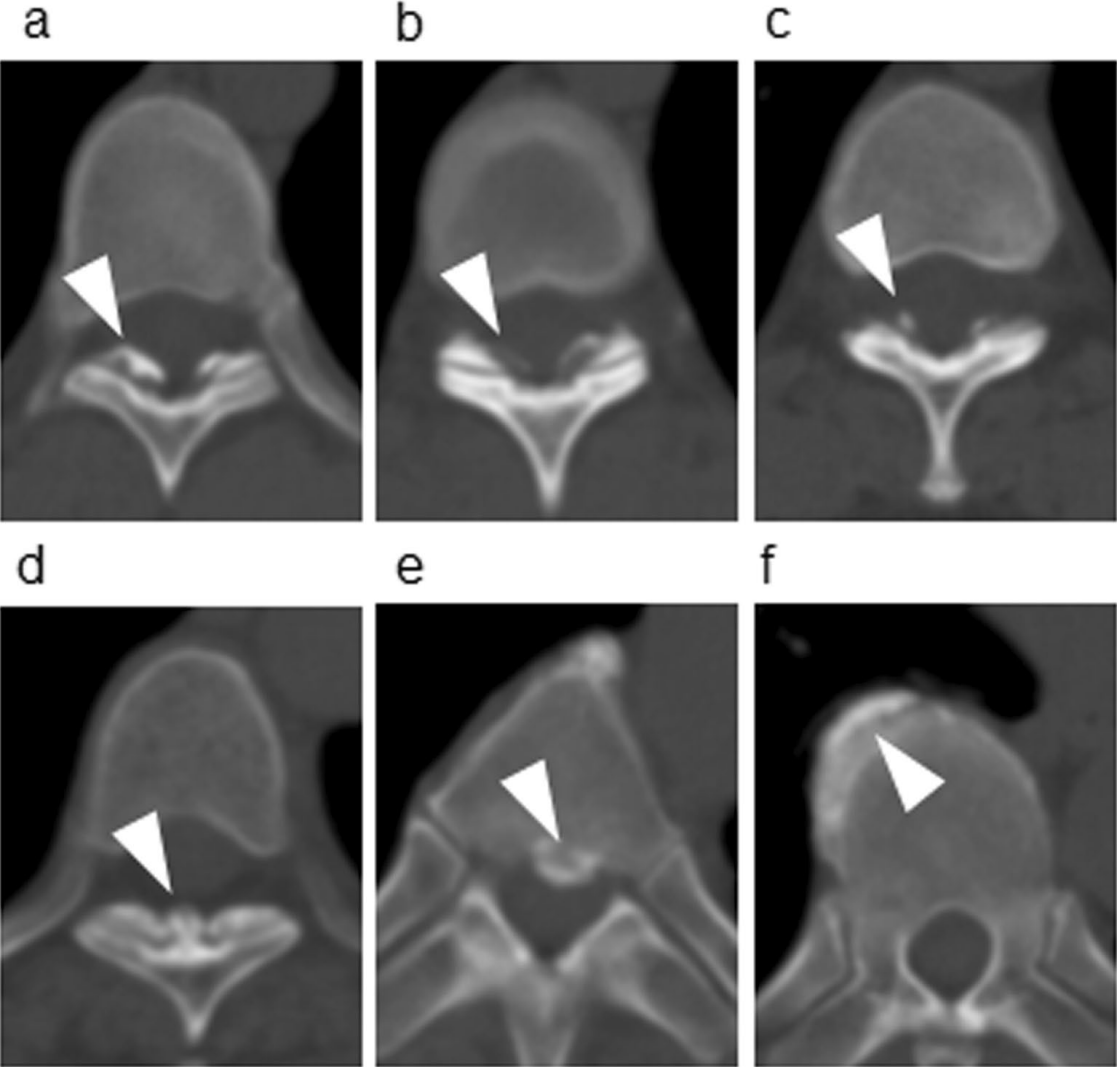
Representative examples of OLF, OPLL, and OALL (indicated using arrowheads) on computed tomography images. (**a)** OLF with thickness ≥ 2 mm. (**b)** OLF with thickness ≥ 1 mm. (**c)** OLF clearly visible at 1 mm thickness. (**d)** A mushroom-shaped OLF localized at the center of the laminae. (**e)** OPLL with thickness ≥ 2 mm. (**f)** OALL located in the paramedian region of the anterior vertebral body with a thickness ≥ 4 mm. *OLF* ossification of the ligamentum flavum, *OPLL* ossification of the posterior longitudinal ligament, *OALL* ossification of the anterior longitudinal ligament.

The presence of OLF was determined according to the method described by Mori et al. with slight modifications^4^. Briefly, OLF was defined as ossification within the ligamentum flavum, excluding facet osteophytes, with a thickness ≥ 1 mm on axial planar images, because the study included asymptomatic subjects with presumably smaller OLF compared to symptomatic patients (Fig. 1a–c). Mushroom-shaped ossification localized in the center of the laminae was also defined as OLF (Fig. 1d). Ossification within the posterior longitudinal ligament with a thickness ≥ 2 mm on axial planar images was defined as OPLL (Fig. 1e)^7^. Ossification of the anterior longitudinal ligament with a thickness ≥ 4 mm on axial planar images, which bridged adjacent vertebral bodies, was defined as OALL, based on minor modifications to a previous method (Fig. 1f)^7^. All CT images were assessed by two board-certified spine surgeons (TE and RF), and disagreements were resolved by consensus to minimize intra- and interobserver bias and errors. Prior to image review, these two readers evaluated the same images of each of OALL, OPLL, and OLF for 20 subjects to determine interobserver agreement. Cohen's kappa coefficient ranged from 0.79 to 0.80, indicating high interobserver agreement.

### Semi-quantitative evaluation of OLF severity

The severity of OLF was calculated using the OLF index^13,15^; briefly, the OLF index was expressed as the sum of the number of intervertebral segments with OLF from the cervical to lumbar spine. (In theory, the minimum OLF index is 0 and the maximum is 23). According to the location of OLF, thoracic OLF index and lumbar OLF index were also evaluated separately. The OLF index in this study was the sum of thoracic and lumbar OLF indices. Prior to image review, two readers (TE and RF) evaluated the same images of 20 subjects to determine interobserver agreement. The intraclass correlation coefficient (ICC) between observers was 0.94, with a 95% confidence interval (CI) of 0.87–0.97, indicating an extremely high interobserver agreement.

### Grouping subjects according to OLF type

The subjects were divided into two groups according to coexisting lumbar OLF. The non-lumbar OLF group (L-OLF[−], n = 169) included subjects with OLF in the thoracic spine but not in the lumbar spine. The lumbar OLF group (L-OLF[+], n = 70) included subjects with OLF in the lumbar spine, and with or without OLF in the thoracic spine.

### Grouping subjects according to OLF severity

Subjects were divided into three groups according to OLF severity based on the OLF index: localized OLF (OLF index ≤ 2, n = 138); intermediate OLF (3 ≤ OLF index ≤ 5, n = 70); and extensive OLF (6 ≤ OLF index ≤ 12, n = 31). The cut-off values for grouping were determined based on our previous study on symptomatic OLF patients^14^. In this previous report, patients with OLF localized between 1 and 2 vertebrae were classified as the localized OLF group, and patients with the OLF-index ≥ 3 were classified as the multilevel OLF group. The OPLL + OLF group was also evaluated. The cut-off values used in this study to classify the intermediate and extensive OLF groups (i.e., the OLF-index: 6) were determined based on the mean OLF-index of the multilevel OLF and OPLL + OLF groups in the previous study.

### Single and multiple regression analysis of factors associated with severity of lumbar OLF

Single regression analysis was utilized to examine the factors associated with the severity of lumbar OLF (i.e., the lumbar OLF index): age, body mass index (BMI), sex, comorbidities, and presence and number of concomitant spinal ligament ossifications. The multiple regression analysis included variables that were statistically significant (*P* < 0.10) in the single regression analysis. However, in the multiple regression analysis, the age, BMI, sex, and coexisting spinal ligament ossification items, including OALL, OPLL, and thoracic OLF, were all included in the analysis, given the purpose of this study.

### Statistical analysis

Data were analyzed using BellCurve for Excel (version 3.10; Social Survey Research Information Co., Ltd., Tokyo, Japan). Data normality was examined using the Shapiro–Wilk test. For nonparametric variables, results are shown as median (minimum and maximum). Differences in continuous variables between the two groups were evaluated using the Mann–Whitney U-test. Differences in proportion between the two groups were evaluated using Fisher's exact test. Statistical significance between two groups was set at *P* < 0.05. Comparisons among the three groups were conducted using the Kruskal–Wallis test for nonparametric variables and Fisher's exact test for proportion. To account for multiple testing, the Bonferroni correction was performed to adjust the statistical significance to *P* < 0.016 (i.e., 0.05/3). The relationships between the factors affecting the severity of OLF were evaluated using multiple regression analysis. Statistical significance was set at *P* < 0.05. The interrater reliability for the presence of spinal ligament ossification was assessed by Cohen's kappa coefficient and for the OLF-index by ICC.

## Results

### Baseline characteristics, disease status, and prevalence of OLF types

Of the 595 asymptomatic subjects for whom CT was available for analysis, 55.5% were men, and the mean age of the subjects was 52.6 years (data not shown). The characteristics of the subjects with OLF are shown in Table 1. The proportion of subjects with OLF at any location of the spine was 40.1% (239/595). The proportion of lifestyle-related diseases (hypertension, diabetes mellitus, and ischemic heart disease) in the subjects with OLF was comparable to that of the general population in Japan^16^. Among all subjects with OLF, 0.4% of subjects had OLF only in the lumbar spine, and 28.8% had OLF in both the thoracic and lumbar spine. No subjects had OLF in the cervical spine.

**Table 1:** Clinical characteristics of the study subjects with OLF.

Variable	Subjects with OLF (n = 239)
Age (years)	54.0 (29–77)
**Age range (%)**	
20–29	0.4 (1)
30–39	7.1 (17)
40–49	29.2 (70)
50–59	31.7 (76)
60–69	24.2 (58)
70–79	7.1 (17)
Male (%)	67.8 (162)
BMI (kg/m^2^)	24.5 (17.6–39.8)
**Comorbidity (%)**	
Hypertension	24.6 (59)
DM	7.9 (19)
IHD	7.5 (18)
Renal disease	0 (0)
**Comorbid OLF (%)**	
OLF	100 (239)
Cervical OLF only	0 (0)
Thoracic OLF only	70.7 (169)
Lumbar OLF only	0.4 (1)
Thoracic + lumbar OLF	28.8 (69)

Data are shown as median (minimum–maximum) or as percentage (number).

*BMI* body mass index, *OLF* ossification of the ligamentum flavum, *DM* diabetes mellitus, *IHD* ischemic heart disease.

### Differences in the proportion of subjects with coexisting spinal ligament ossification according to the presence of lumbar OLF

**Figure 2:**
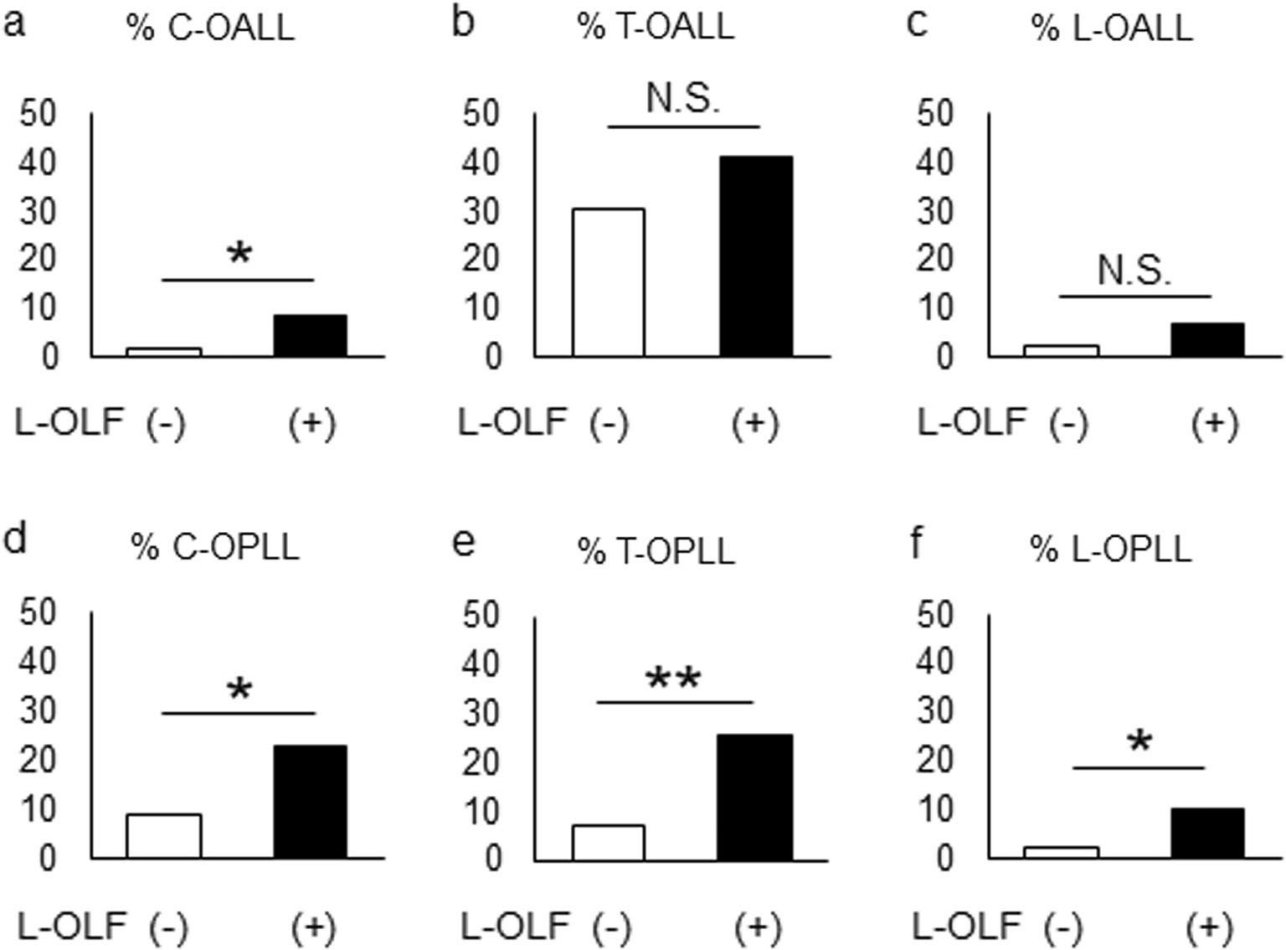
Comparison of comorbidity of OALL and OPLL in subjects with lumbar OLF [L-OLF(+)] or without lumbar OLF [L-OLF(−)]. *P < 0.01, **P < 0.001, *N.S.* not significant. *OALL* ossification of the anterior longitudinal ligament, *OPLL* ossification of the posterior longitudinal ligament, *OLF* ossification of the ligamentum flavum, *C* cervical, *T* thoracic, *L* lumbar.

To compare the clinical backgrounds of subjects with and without lumbar OLF, we divided the subjects with OLF into the L-OLF(−) and L-OLF(+) groups. The severity of OLF, calculated using the OLF index, was significantly higher in the L-OLF(+) group than in the L-OLF(−) group (*P* < 0.001) (Table 2). The proportion of subjects with coexisting C-OALL, C-OPLL, T-OPLL, and L-OPLL in the L-OLF(+) group was also significantly higher than that in the L-OLF(−) group (Fig. 2).

**Table 2:** Comparison of clinical characteristics between subjects with or without lumbar OLF.

Variable	L-OLF( −) (n = 169)	L-OLF( +) (n = 70)	*P* value
Age (years)	54.0 (30–77)	53.5 (29–77)	0.69
**Age range (%)**			
20–29	0	1.4	NA
30–39	8.2	4.2	0.27
40–49	28.9	30.0	0.87
50–59	31.9	31.4	0.93
60–69	23.6	25.7	0.73
70–79	7.1	7.1	0.99
BMI (kg/m^2^)	24.1 (17.6–39.8)	25.1 (18.4–36.9)	0.10
Male (%)	66.9	70.0	0.63
**Comorbidity (%)**			
Hypertension	25.4	22.8	0.53
DM	8.2	7.1	0.50
IHD	5.9	11.4	0.25
Renal disease	0	0	NA
OLF index	2.0 (1–7)	4.0 (1–12)	< 0.001

Data are shown as median (minimum–maximum) or as percentage.

*BMI* body mass index, *OLF* ossification of the ligamentum flavum, *L* lumbar, *DM* diabetes mellitus, *IHD* ischemic heart disease, *NA* not available.

### Association between ossification severity and coexisting lumbar OLF

**Figure 3:**
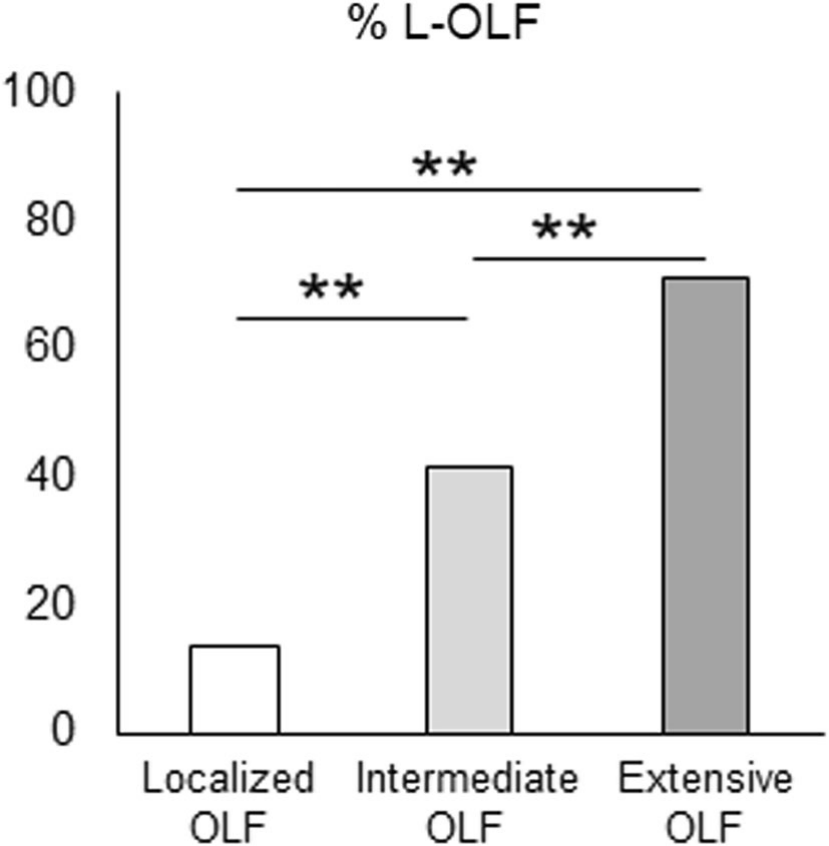
Comparison of comorbidity of lumbar OLF among the three OLF categories (localized OLF, OLF index ≤ 2; intermediate OLF, 3 ≤ OLF index ≤ 5; extensive OLF, 6 ≤ OLF index ≤ 12). *P < 0.01, **P < 0.001. *OPLL* ossification of the posterior longitudinal ligament, *OLF* ossification of the ligamentum flavum, *L* lumbar.

We examined the impact of OLF severity in the entire spine on the development of lumbar OLF by classifying subjects according to the OLF index into localized, intermediate, and extensive OLF groups. The prevalence of spinal ligament ossification in each group is shown in Table 3. The proportions of subjects with coexisting C-OALL and T-OPLL in the extensive OLF group were significantly higher than those in other groups (*P* < 0.016). The proportion of subjects with coexisting L-OPLL in the extensive OLF group was higher than that in the localized OLF group (*P* < 0.016). The proportions of subjects with coexisting T-OALL, L-OALL, and C-OPLL in the extensive OLF group were higher than those in the other groups; however, the differences were not significant. The proportion of subjects with coexisting L-OLF increased significantly as the OLF index grade increased (*P* < 0.001) (Fig. 3).

**Table 3:** Clinical characteristics and rate of comorbid spinal ligament ossification in subjects classified by the severity of OLF.

Variable	Localized OLF (n = 138)	Intermediate OLF (n = 70)	Extensive OLF (n = 31)
Age (years)	54.5 (30–77)	51.0 (29–74)	58.0 (40–76)
**Age range (%)**			
20–29	0	1.4	0
30–39	7.9	8.5	0
40–49	26.8	30.0	38.7
50–59	31.8	38.5	16.1
60–69	25.3	18.5	32.2
70–79	7.9	2.8	12.9
BMI (kg/m^2^)	24.0 (17.6–39.8)	25.3 (18.4–35.5)	23.8 (20.3–36.9)
Male (%)	62.3	71.4	83.8
**Comorbidity (%)**			
Hypertension	24.6	27.1	19.3
DM	9.4	8.5	0
IHD	7.2	7.1	9.6
Renal disease	0	0	0
**Comorbid spinal ligament ossification (%)**			
C-OALL	2.1	1.4	16.1*^,†^
T-OALL	31.8	31.4	48.3
L-OALL	2.8	2.8	9.6
C-OPLL	11.5	12.8	19.3
T-OPLL	9.4	10.0	32.2*^,†^
L-OPLL	2.8	4.2	12.9*

Data are shown as median (minimum–maximum) or as percentage. The three groups are as follows: localized OLF (OLF index ≤ 2); intermediate OLF (3 ≤ OLF index ≤ 5); and extensive OLF (6 ≤ OLF index ≤ 12).

*P < 0.016 versus localized OLF group.

^†^*P* < 0.016 versus intermediate OLF group.

*OLF* ossification of the ligamentum flavum, *OALL* ossification of the anterior longitudinal ligament, *OPLL* ossification of the posterior longitudinal ligament, *BMI* body mass index, *DM* diabetes mellitus, *IHD* ischemic heart disease, *C* cervical, *T* thoracic, *L* lumbar.

### Factors associated with severity of OLF in the lumbar spine

Regression coefficients (β) and 95% CI for regression analyses examining the factors associated with the lumbar OLF index are presented in Table 4. In the single regression analysis, the lumbar OLF index was significantly associated with the co-existence of C-OALL, C-OPLL, T-OPLL, L-OPLL, and thoracic OLF index (*P* < 0.05) (data not shown). In the multiple regression analysis, lumbar OLF index was significantly associated with thoracic OLF index and with the co-existence of thoracic and lumbar OPLL (thoracic OLF index: β, 0.07; 95% CI 0.01–0.13; co-existence of thoracic OPLL: β, 0.61; 95% CI 0.26–0.96; co-existence of lumbar OPLL: β, 0.62; 95% CI 0.04–1.19).

**Table 4:** Multiple regression analysis for factors associated with lumbar OLF index.

Independent variables	β	95% CI	*P* value
Age (years)	− 0.00	− 0.01 to 0.00	0.36
BMI (kg/m^2^)	− 0.00	− 0.03 to 0.03	0.97
Sex (Male)	− 0.01	− 0.26 to 0.24	0.92
**Co-existence of**			
C-OALL	0.28	− 0.35 to 0.91	0.38
T-OALL	0.07	− 0.19 to 0.35	0.57
L-OALL	0.12	− 0.49 to 0.73	0.69
C-OPLL	0.27	− 0.06 to 0.61	0.11
T-OPLL	0.61	0.26–0.96	< 0.001
L-OPLL	0.62	0.04–1.19	0.03
T-OLF	0.10	− 1.69 to 1.90	0.90
Thoracic OLF index	0.07	0.01–0.13	0.01

*β* regression coefficient, *CI* confidence interval, *BMI* body mass index, *OLF* ossification of the ligamentum flavum, *OALL* ossification of the anterior longitudinal ligament, *OPLL* ossification of the posterior longitudinal ligament, *C* cervical, *T* thoracic, *L* lumbar.

## Discussion

The present study revealed that the severity of lumbar OLF was associated with the severity of OLF in the thoracic spine. Among all asymptomatic subjects with OLF, the proportion with OLF in the lumbar spine was 29.2%. This study was designed to minimize selection bias by using a large dataset of single-center medical check-ups, which is common in Japan; many healthy subjects, including doctors, nurses, administrative staff, and community residents, underwent this medical check-up. This study also showed that the co-existence of thoracic and lumbar OPLL was associated with the severity of lumbar OLF, thus identifying the propensity for ossification of spinal ligaments in subjects who develop lumbar OLF. Given that the pathogenesis of OLF has been poorly studied compared with that of OPLL, categorizing the patient characteristics associated with ossification development provides a good basis for exploring its pathogenesis.

Our results suggest that OLF can be caused by the ossification tendency of the entire spine and not just by local mechanical stimulation. This suggestion is reinforced by our findings that (1) a number of intervertebral segments of lumbar OLF was associated with that of thoracic OLF (Table 4), and (2) subjects with lumbar OLF tended to have a high rate of coexisting cervical OALL (5 times), cervical OPLL (2.5 times), thoracic OPLL (3.5 times), and lumbar OPLL (4 times) than those without lumbar OLF (Fig. 2). Considering that OLF is most likely to be observed in the thoracolumbar region^7^, the conventional hypothesis that mechanical stress is a factor in the development of OLF has some validity^8–10^. Recently, however, it was shown that patients with OPLL in the thoracic spine have a higher degree of obesity and a tendency toward diffuse heterotopic ossification of the entire spine, including OLF, compared to those with OPLL localized in the cervical spine^12,13,17^. In addition, patients with multilevel OLF tend to be more obese than those with localized OLF^14^. Leptin, a type of adipokine which originates from adipose tissue, acts directly on osteoblasts and chondrocytes to promote bone formation^18–20^. Insulin-like growth factor-1 (IGF-1), an anabolic hormone expressed in most tissues, promotes osteoblast differentiation and calcification^21–23^. Compared to the thoracolumbar region, the thoracic spine suffers less mechanical stress due to the support of the rib cage; thus, these findings suggest that spinal ligament ossification in multiple regions, including the thoracic spine, can be caused by systemic bone metabolism due to intrinsic (humoral and genetic) factors, as well as by mechanical stimulation.

Genetic factors may be involved in the pathogenesis of OLF. Although genetic information on OLF is very limited compared to OPLL, previous reports suggested that COL6A1, identified as an OPLL susceptibility gene in Japanese^24^, is a common susceptibility gene for both OLF and OPLL in the Han Chinese population^25,26^. Further gene discovery research targeting OLF is warranted, but we should note that OPLL and OLF often coexist^11–13^, making it difficult to strictly distinguish whether a gene is associated with OPLL or OLF.

The finding that BMI is not an independent risk factor for the severity of lumbar OLF was contrary to our expectations. It was expected that the mean BMI of OLF subjects with coexisting thoracic OPLL would be higher than those without coexisting thoracic OPLL (27 kg/m^2^ vs. 24 kg/m^2^) (data not shown); however, it was unexpected that the mean BMI was comparable among the localized OLF, intermediate OLF and extensive OLF groups (Table 3). A previous study showed that the mean BMI of patients with multilevel OLF over the entire spine was > 28 kg/m^2^.^14^ This discrepancy with the previous study on symptomatic patients could be due to the inclusion of asymptomatic subjects only in this study; a large number of healthy subjects were included in this study.

In the present study, 40% of all subjects had OLF in the thoracic spine and 11% had OLF in the lumbar spine. A wide range for the prevalence of OLF has been reported, from 3.6 to 63.9%^1–4,7,27–29^. This wide range is likely due to the diagnostic modalities of assessing for presence of spinal ligament ossification; the prevalence of OLF in the two studies using lateral radiographs was as low as 3.6–6.2%^27,28^, whereas the prevalence in the four studies using CT was much higher, ranging from 12 to 63.9%^3,4,7,29^. Mori et al. defined OLF with a thickness ≥ 3 mm or < 3 mm but distinctly identifiable as OLF-positive^4^. They also included mushroom-type OLF located in the center of the lamina. Our result was almost comparable to the proportion of subjects with OLF in the thoracic spine reported by Mori et al. (36%). On the other hand, Fujimori et al. defined relatively large OLF with a thickness ≥ 4 mm as OLF-positive, but it was unclear whether they included mushroom-shaped OLF^7^. They reported that 12% of subjects had OLF in the thoracic spine and 0.6% had OLF in the lumbar spine. Thus, we concluded that the prevalence of OLF is likely to vary depending on criteria for both type and size of ossification.

This study had some limitations. First, as this was a cross-sectional study, the association factors identified do not indicate the cause of OLF. Second, the sample size of the extensive OLF group which we focused on was relatively small. Considering that localized OLF often develops in the thoracic spine, a multicenter nationwide study with a larger sample size is needed to validate our results. Third, this study was based on data from Japanese participants, and hence, may not necessarily apply to other nations. Finally, the majority of the 12,390 subjects who underwent physical examinations did not undergo CT scans or underwent CT of either the trunk or the cervical spine. In addition, the radiological findings were not evaluated in a blinded-fashion. Thus, the risk of selection bias and information bias cannot be eliminated.

In summary, this study revealed that subjects with multilevel OLF in the lumbar spine are likely to have multilevel OLF over the entire spine. Patients with lumbar OLF are potentially a distinct subgroup of patients with a strong tendency to ossification of the entire spinal ligament. These suggested that their ossification can be caused by systemic bone metabolism associated with intrinsic factors as well as by mechanical factors. In the future, controlling systemic metabolic abnormalities may prevent their ossification from worsening and they also appear to be an important patient subgroup for identifying aggravating factors in spinal ligament ossification.

## Data Availability

The dataset of this study is not publicly available. However, on reasonable request, derived data supporting the findings of this study are available from the corresponding author after approval from the Ethical Committee of the Hakodate Central Hospital and Hokkaido University Hospital.
